# Window Period Prophylaxis for Children Exposed to Tuberculosis, Houston, Texas, USA, 2007–2017

**DOI:** 10.3201/eid2503.181596

**Published:** 2019-03

**Authors:** Andrea T. Cruz, Jeffrey R. Starke

**Affiliations:** Baylor College of Medicine, Houston, Texas, USA;; Children’s Tuberculosis Clinic at Texas Children’s Hospital, Houston

**Keywords:** directly observed therapy, directly observed preventive therapy, isoniazid, tuberculin skin test, tuberculosis exposure, window prophylaxis, window period prophylaxis, tuberculosis, prophylaxis, safety, retrospective study, pediatric, children, asymptomatic, TST conversion, mycobacteria, tuberculosis and other mycobacteria, bacteria, *Mycobacterium tuberculosis*, United States, Houston, Texas, adverse events, acid-fast bacilli smear, cohabitation

## Abstract

In this retrospective study, we assessed the safety of window period prophylaxis and proportion of tuberculin skin test (TST) conversions in children <5 years of age who were exposed to an adult with tuberculosis disease during 2007–2017. Children included in this study had unremarkable examination and chest radiograph findings and negative test results for TB infection. In total, 752 children (41% cohabitating with the index patient) received prophylaxis during the window period, usually directly observed therapy with isoniazid. Hepatotoxicity and tuberculosis disease did not develop in any child. TST conversion occurred in 37 (4.9%) children and was associated with the index patient being the child’s parent (odds ratio 3.2, 95% CI 1.2–8.2). TST conversion was not associated with sputum smear results, culture positivity, or cohabitation. Thresholds for initiation of window prophylaxis in exposed young children should be low given the safety of medication and difficulties with risk stratification.

In 2017, tuberculosis (TB) disease was diagnosed in >9,000 persons in the United States (*1*). Each time a person (the index patient) receives a diagnosis of potentially contagious TB, health departments query that patient to determine recent contacts; this practice enables active surveillance and identification of persons who would benefit from evaluation and possibly treatment. Young children have a high rate of TB disease development shortly after infection (*2*). Prechemotherapy studies show that disease develops in 40%–50% of recently infected children <1 year of age and 25% of those 1–2 years of age, and miliary or meningeal TB develops in 25% of the children with TB disease <1 year of age and 20% of those 1–2 years of age. The rates of disease development gradually decreased with age among 3–5-year-olds (*2*).

US guidelines recommend that all children <5 years of age who are exposed to a person with potentially contagious TB be assessed for TB disease. This evaluation should include symptom screening, physical examination, and chest radiography, regardless of symptoms. In addition, children should receive a test of infection (either a tuberculin skin test [TST] or the interferon-γ release assay [IGRA]). If children have an examination and chest radiograph with unremarkable results and an initial negative test of infection, treatment is offered during what is termed the window period (i.e., the time it can take for a TST or IGRA to become positive after a person has shared air with another person with contagious TB). This window prophylaxis therapy is continued until a second test of infection performed 8–12 weeks after the last exposure determines definitively if infection has occurred (*3*).

Given the safety profile of TB medications in children (*4*,*5*) and the risk for serious disease rapidly developing, treatment during this window is considered highly beneficial for young children. However, although window prophylaxis has been used for decades, its safety and effectiveness have not been assessed. The goals of this study were to evaluate safety and tolerability of window prophylaxis in young children and determine the proportion and predictors of developing TB infection or disease in these exposed children.

## Methods

We performed a retrospective cohort study of children <5 years of age referred to pediatric TB clinics in Houston, Texas, USA, during 2007–2017. Our clinic (Children’s Tuberculosis Clinic at Texas Children’s Hospital, Houston, Texas, USA) is the main referral source for children seen in 12 counties of the greater Houston metropolitan area (population ≈7 million), where TB incidence is twice the national average (*6*). We included all infants <6 months of age who had recent contact with an index TB patient, as well as all other children <5 years of age who had recent contact with an index patient and initial negative diagnostic test results (TST <5 mm or negative IGRA), no symptoms of TB disease, and an unremarkable 2-view chest radiograph and who were started on window prophylaxis. We excluded children whose contacts were later determined to not have TB or to have solely extrapulmonary TB.

Children are within the window period until 8–12 weeks after contact with the index patient has ceased, either by physical separation or effective treatment of the index patient (generally reflected by 3 consecutive negative results on acid-fast bacilli [AFB] sputum smear testing) (*7*). Health department workers performed a TST or obtained blood for IGRAs in the home of the child as soon as the child’s exposure was recognized and 8–12 weeks after contact was broken. In the interim, isoniazid window prophylaxis was offered to exposed children twice weekly (20–30 mg/kg/dose, maximum 900 mg/d) under directly observed preventive therapy (DOPT) in the child’s home by health department workers, as previously described (*8*). If the index case isolate was resistant to isoniazid or isoniazid was contraindicated, healthcare workers administered rifampin daily (10–20 mg/kg/dose, maximum dose 600 mg/d). Children were seen in the clinic at the initiation of therapy and every 1–2 months thereafter. We abstracted medical records for demographic variables, epidemiology and microbiology results for the index patient, medication regimens, and adverse events (AEs). We obtained institutional review board approval from Baylor College of Medicine (Houston, Texas, USA).

The primary outcome was the proportion of families reporting AEs for their children while they were on window prophylaxis. All children were assessed for signs and symptoms of hepatotoxicity at each DOPT medication visit. Children with abdominal pain, vomiting, anorexia, icterus, or weight loss had their medications withheld pending clinical and laboratory evaluation. Otherwise healthy asymptomatic children did not have baseline or serial liver testing performed. We used the National Cancer Institute guidelines to grade AE severity (*9*). The secondary outcomes were the proportion of infants with an initially negative test of infection in which TB infection or disease subsequently developed while on treatment and the epidemiologic factors associated with TST or IGRA conversion. We considered TST results of ≥5 mm of induration and QuantiFERON (QIAGEN, https://www.qiagen.com) results ≥0.35 international units/mL positive (*10*,*11*). We performed a priori analyses to evaluate test conversion in children <2 years and ≥2 years of age because children in the first 2 years of life are at the highest risk for disease progression with untreated TB infection (*2*). We compared proportions using odds ratios (ORs) with 95% CIs and expressed continuous variables as medians with interquartile ranges (IQRs). We used Stata 14 (StataCorp, https://www.stata.com) for analyses.

## Results

### Demographics

#### Pediatric Demographics

During the study period, 841 children from 12 health departments in the greater Houston area were seen for TB exposure. In total, 89 (10.6%) children were not started on window prophylaxis; 76 of these children had already had 2 negative tests of infection separated by 8–12 weeks, 8 (1%, 8/841) had familial refusal, 4 immediately moved out of the area, and 1 child had an index case that was multidrug-resistant (MDR) TB. The remaining 752 (89.4%) children initiated window prophylaxis (Table 1). No differences were found between children who did and did not receive window prophylaxis regarding age, race/ethnicity, or sex of the child or AFB sputum smear positivity of the index patient.

**Table 1:** Characteristics of 752 children exposed to index tuberculosis patients, index patients, and index case *Mycobacterium tuberculosis* isolates, Houston, Texas, USA, 2007–2017

Characteristic	Value*
Demographics	
Age, y, median (interquartile range)	2.4 (1.2–3.6)
Sex	
F	380 (50.5)
M	372 (49.5)
Race/ethnicity	
Hispanic	493 (65.6)
Black	141 (18.8)
Asian	78 (10.4)
White	30 (4.0)
Biracial	10 (1.3)
Residing in home of index patient	
Yes	311 (41.4)
No	441 (58.6)
Index patient microbiology	
Acid-fast bacilli smear positive	513 (68.2)
Acid-fast bacilli culture positive	680 (90.4)
Index isolate drug susceptibilities, n = 680	
Isoniazid and rifampin susceptibility	635 (93.4)
Isoniazid resistance†	32 (4.7)
Isoniazid and rifampin resistance	13 (1.9)
Rifampin monoresistance	1 (0.1)
Index patient sample used for culture	
Sputum	637 (84.7)
Bronchoalveolar lavage	93 (12.4)
Lung biopsy	11 (1.5)
Pleural fluid	11 (1.5)

*Values are no. (%), except where specified. Percentages might not sum up to 100% because of rounding. †Seven isolates were resistant to isoniazid and streptomycin, and 5 were resistant to isoniazid and ethambutol; the remainder of isolates had isoniazid monoresistance.

#### Index Patient Demographics

A total of 483 unique index patients (median age 44 [IQR 30–59] years) had preschool-aged contacts. In 311 (41.4%) of 752 instances, the index patient lived in the same home as the child. The most common relationship of the index patient to the child was grandparent or great-grandparent (37.8%, 284), followed by aunt or uncle (25.8%, 194), parent (15.8%, 119), cousin (6.5%, 49), sibling (0.9%, 7), other relative (2.4%, 18), babysitter (1.6%, 12), and other nonrelative (9.2%, 69).

### Regimens

The most common medication used was isoniazid (730/752, 97.1%), followed by rifampin (20/752, 2.7%); 2 children whose index patient had MDR TB received ethambutol and pyrazinamide. For 17 (2.3%) children, therapy was changed after drug susceptibilities became available for the source case isolate; in these cases, the drug was changed to rifampin for 8 children and ethambutol and pyrazinamide for 2 children. For 7 children, isoniazid was stopped and no other drug begun because the second TST result was by then available and negative. These regimen changes occurred a median of 6 (IQR 2–8) weeks into therapy. Two (0.3%) children did not complete therapy: 1 child whose family refused medications and 1 child whose family moved out of the area before the second TST was performed.

### Safety

No child had hepatotoxicity or TB disease progression while receiving window prophylaxis. The presence of any AE was rare (7/752, 0.9%). Rash developed in 2 patients; emesis in 2 patients; and diarrhea, weakness, and angioedema each in 1 patient. All 7 of those patients were taking isoniazid. Two AEs (angioedema, weakness) were grade 2, and the remainder were grade 1. The median time to development of an AE was 5 (IQR 2–6) weeks. TB treatment for the 7 children with AEs was changed to rifampin, which was well tolerated for the remainder of window prophylaxis. Children received therapy almost exclusively (744/752, 98.9%) under direct observation and for a median of 9 (IQR 7–12) weeks.

### Conversion of Test of Infection

TSTs were used for initial and subsequent testing in 749 (99.6%) children. The median time between the first and definitive TST was 73 (IQR 63–90) days. This time interval did not differ between children residing (77 days) and not residing (78 days) in the same household as the index patient (p = 0.47) or between children younger (83 days) or older (78 days) than 6 months (p = 0.09).

Tests of infection converted from negative to positive in 37 (4.9%) children a median of 11 weeks after the initial test of infection. No differences were observed in TST conversion between children <2 years and ≥2 years of age (p = 0.11). The median TST induration in these 37 children was 12 (IQR 10–15) mm. TST conversion was more common when the index patient was young or the child’s parent. No microbiological parameters (e.g., AFB smear and culture status) or epidemiologic factors (e.g., residence in same or different household) were associated with TST conversion (Table 2).

**Table 2:** Association between epidemiologic and microbiologic variables and tuberculin skin test conversion among children exposed to index tuberculosis patients, Houston, Texas, USA, 2007–2017

Variable	Tuberculin skin test conversion*		Value†
	Converted, no. (%), n = 37	Not converted, no. (%), n = 715	
Child demographics			
Age, y			
0–<1	4/154 (2.6)	150/154 (97.4)	Referent
1–<2	8/164 (4.9)	156/164 (95.1)	1.9 (0.6–6.5)
2–<5	25/434 (5.8)	409/434 (94.2)	2.3 (0.8–6.7)
Sex			
F	21/380 (5.5)	359/380 (94.5)	Referent
M	16/372 (4.3)	356/372 (95.7)	0.8 (0.4–1.5)
Race/ethnicity			
Hispanic	24/493 (4.9)	469/493 (95.1)	Referent
Black	5/141 (3.5)	136/141 (96.5)	0.7 (0.3–1.9)
Asian	4/78 (5.1)	74/78 (94.9)	1.1 (0.4–3.1)
Other	4/40 (10)	36/40 (90)	2.1 (0.7–6.6)
Index patient demographics			
Age, y	37	46	p = 0.002
Relationship to child			
Grandparent	8/284 (2.8)	276/284 (97.2)	Referent
Parent	10/119 (8.4)	109/119 (91.6)	3.2 (1.2–8.2)
Other relative	13/255 (5.1)	242/255 (94.9)	0.7 (0.3–1.8)
Nonrelative	6/94 (6.4)	88/94 (93.6)	0.9 (0.3–2.8)
Household member of child			
No	17/441 (3.9)	424/441 (96.1)	Referent
Yes	20/311 (6.4)	291/311 (93.6)	1.7 (0.9–3.3)
Index patient microbiology			
Sample type cultured			
Nonsputum‡	3/115 (2.6)	112/115 (97.4)	Referent
Sputum	34/637 (5.3)	603/637 (94.7)	2.1 (0.6–7.0)
Acid-fast bacilli stain result			
Negative	8/239 (3.3)	231/239 (96.7)	Referent
Positive	29/513 (5.7)	484/513 (94.3)	1.7 (0.8–3.8)
Acid-fast bacilli culture result			
Negative	3/72 (4.2)	69/72 (95.8)	Referent
Positive	34/680 (5.0)	646/680 (95.0)	1.2 (0.4–4.0)
Isoniazid-susceptible isolate§	30/635 (4.7)	605/635 (95.3)	Referent
Isoniazid-resistant isolate§	4/45 (8.9)	41/45 (91.1)	2.0 (0.7–5.9)

*Percentages reflect within-row frequencies and might not sum up to 100% because of rounding. †Values are odds ratios (95% CI) except where indicated. ‡Bronchoalveolar lavages, lung biopsies, and pleural fluid cultures. §Isoniazid resistance documented only in microbiologically confirmed cases.

In total, 35 (94.6%) of 37 children with TB infection completed therapy; for 1 child, the family opted to stop isoniazid treatment after 2 months, and for the other, whose source case was MDR TB, treatment with ethambutol and pyrazinamide was stopped after abdominal pain developed (hepatic transaminases for this patient were within reference ranges). To the best of our knowledge, TB disease did not develop in any child with TB exposure or with a TB infection with a total of 4,466 (median 5.7 [IQR 3.7–7.6]) person-years of follow-up.

## Discussion

We found that most families accepted their young children being treated for TB exposure. Therapy was initiated and completed for most children, most of whom were treated with twice-weekly DOPT with isoniazid. This treatment was safe; no child developed hepatotoxicity. Progression from exposure to infection was uncommon and not associated with epidemiologic factors typically associated with transmission of *M. tuberculosis*. Health department workers predominantly used TSTs for testing and isoniazid for window prophylaxis and only infrequently used IGRA and rifamycin-based regimens.

Prior studies have shown that up to 50% of children do not begin or complete isoniazid therapy for TB infection when children or other family members administer the drug (*5*,*12*,*13*). In contrast, families of TB-exposed children felt comfortable directly observing the administration of medication to their young, asymptomatic children who had unremarkable examination findings and chest radiograph results and negative tests of infection. Their acceptance of this therapy might reflect them having known a symptomatic contact (*14*) but, we hope, was also supported by our counseling. Directly observed therapy (DOT), which removes barriers to medication acquisition and administration, is often used to optimize adherence (*13*) and enables close monitoring for drug toxicities. We had a low threshold to change drug regimens in the event of any AE to facilitate continuation of window prophylaxis.

In this study, we did not find an association between TST conversion and AFB sputum smear status or cohabitation with the index patient, both found to be risk factors for acquisition of TB infection in other studies (*15*,*16*). Several factors could help explain this discrepancy. First, baseline smear positivity is not an accurate proxy of the degree of infectiousness. Other factors, such as intensity of exposure, room air exchanges, radiographic findings, sputum viscosity, and aerosolization of viable bacteria, also affect transmission (*17*,*18*). In fact, some data suggest that adults with high concentrations of bacteria in their smears might not transmit TB efficiently because they are too weak to produce a vigorous cough (*19*). Second, infected persons are not equally contagious throughout the natural history of infection, and discerning when during the index patient’s course of infection the exposure to the child occurred is often difficult. Third, children were defined as household contacts if they resided in the same home as the index patient. However, some children spent most of their waking hours in another home cared for by a nonparent index patient, in which case, the child was defined as a nonhousehold contact. A rigid definition of what constitutes a household contact lacks epidemiologic meaning (*20*). Another finding from our study was that epidemiologic and microbiological factors were inadequate to risk stratify the children at higher risk of progressing from TB exposure to infection. As such, we feel that all exposed young children need to be evaluated with a physical examination, chest radiograph, and a test of infection and that all families should be offered window prophylaxis for their children. Of note, a TB exposure risk scoring system has been validated for children in settings of TB hyperendemicity (*21*). Although this system was not validated in low-incidence settings, such as the United States, discussing some of the scoring system variables associated with increased risk for exposure (maternal TB, sleep proximity, and duration of exposure) with families reluctant to initiate therapy might be useful.

Our primary diagnostic test for exposed children was TSTs, and treatment was primarily with isoniazid. The decision to use this testing and treatment strategy was driven by several considerations: a paucity of data on IGRAs and other treatment regimens in young children, ease of administration, and cost. The ability to place and read a TST in the home, rather than referring a child to a laboratory for an IGRA, has cost and convenience advantages for families. Given the large number of children evaluated for TB annually, using lower cost tests of infection and medications enabled health departments to provide services to more patients. Video DOT (i.e., the secure uploading and transmission of videos to the health department showing the child taking medication) enables remote monitoring of adherence and toxicity and is less costly than standard DOT (*22*). Rifampin is considerably more expensive than isoniazid, and data do not support giving rifampin less frequently than daily, making DOPT with this drug difficult and expensive. The safety and efficacy of giving window prophylaxis with either medication by DOPT or by family member administration has not been compared in any studies.

Routine use of rifampin for window prophylaxis is reasonable in certain communities where isoniazid-resistant *M. tuberculosis* isolates are highly prevalent. In the United States, ≈8% of isolates are isoniazid resistant and rifampin susceptible (*6*). In our clinic, efficient communication with our health department partners facilitated prompt changing of regimens on the basis of source case drug susceptibility results. Another possible use for rifampin may be for exposed young infants, where concern about TST anergy can result in children receiving therapy for much longer than 8–12 weeks. For these children, 4 months of rifampin might be provided before physicians feel comfortable relying on the definitive TST.

For the children whose test of infection converted to positive, indicating TB infection, we chose to continue twice-weekly isoniazid and complete the 9-month regimen. Considering the data on safety, effectiveness, and adherence to 4-month regimens of daily rifampin (among children) and 3-month regimens of weekly isoniazid and rifapentine (for children ≥2 years of age) (*4*,*23*), changing to 1 of these shorter regimens seems reasonable. Whether the duration of either of these treatments could be shortened in the event of previous isoniazid window prophylaxis is unclear, but giving the infected child the full course of either regimen is most reasonable.

This study had some limitations. Not all variables were documented for all children. Children could have moved outside the area and TB disease could have developed thereafter. Our study was not powered to identify exposure risk factors for TST conversion; with only a 4.9% conversion rate, we might not have had enough cases to adequately assess the influence of certain putative risk factors. Almost all children had serial TSTs and not IGRAs; thus, we cannot rule out the possibility of boosting in the children who had TST conversions. Radiographic findings for the index patient were not uniformly available from local health departments; as such, we could not correlate cavitary disease or other forms of multibacillary disease with the risk for infection in children. Quantifying the duration of exposure was impossible; instead, we used living within the same or different household as a proxy. Timing of when contact was broken for children who were no longer around the contagious person was subject to recall bias. The study was not powered to evaluate efficacy of treatment. However, given the age of the children who had TST conversions (*2*), we estimate that 4–5 children would have progressed to TB disease if untreated. Last, because window prophylaxis is a recommended intervention, performing a randomized controlled trial comparing treatment and no treatment would have been unethical. The administration of window prophylaxis potentially could have prevented the establishment of TB infection in some children; thus, our study potentially underestimates the effect of window prophylaxis in the prevention of infection and disease.

In conclusion, ≈5% of TB-exposed children given prophylaxis progressed to TB infection, and available epidemiologic or microbiological data could not accurately identify which children might receive more benefit from window prophylaxis. However, given the safety of the medications and the acceptability of treatment by families, the benefits of therapy greatly outweigh the risks in this vulnerable patient population.

## References

[1] Stewart RJ, Tsang CA, Pratt RH, Price SF, Langer AJ. Tuberculosis - United States, 2017. MMWR Morb Mortal Wkly Rep. 2018;67:317–23. 10.15585/mmwr.mm6711a2 29565838 PMC5868206

[2] Marais BJ, Gie RP, Schaaf HS, Hesseling AC, Obihara CC, Starke JJ, et al. The natural history of childhood intra-thoracic tuberculosis: a critical review of literature from the pre-chemotherapy era. Int J Tuberc Lung Dis. 2004;8:392–402. 15141729

[3] American Academy of Pediatrics. Tuberculosis. In: Kimberlin DW, Brady MT, Jackson MA, Long SS, editors. Red Book: 2018 report of the Committee on Infectious Diseases. 31st ed. Itasca (IL): American Academy of Pediatrics; 2018. p. 852.

[4] Diallo T, Adjobimey M, Ruslami R, Trajman A, Sow O, Obeng Baah J, et al. Safety and side effects of rifampin versus isoniazid in children. N Engl J Med. 2018;379:454–63. 10.1056/NEJMoa1714284 30067928

[5] Cruz AT, Starke JR. Completion rate and safety of tuberculosis infection treatment with shorter regimens. Pediatrics. 2018;141:e20172838. 10.1542/peds.2017-2838 29363561

[6] Division of Tuberculosis Elimination, National Center for HIV/AIDS, Viral Hepatitis, STD, and TB Prevention, Centers for Disease Control and Prevention. Reported tuberculosis in the United States, 2016. 2017 [cited 2018 Oct 9]. https://www.cdc.gov/tb/statistics/reports/2016/pdfs/2016_Surveillance_FullReport.pdf

[7] Jensen PA, Lambert LA, Iademarco MF, Ridzon R; CDC. Guidelines for preventing the transmission of *Mycobacterium tuberculosis* in health-care settings, 2005. MMWR Recomm Rep. 2005;54(RR-17):1–141. 16382216

[8] Cruz AT, Starke JR. Twice-weekly therapy for children with tuberculosis infection or exposure. Int J Tuberc Lung Dis. 2013;17:169–74. 10.5588/ijtld.12.0641 23317951

[9] National Cancer Institute, National Institutes of Health, US Department of Health and Human Services. Common toxicity criteria (CTC) version 2.0. 1999 Apr 30 [cited 2018 Oct 3]. https://ctep.cancer.gov/protocolDevelopment/electronic_applications/docs/ctcv20_4-30-992.pdf#search=&ldquo;revised common toxicity criteria&rdquo

[10] Targeted tuberculin testing and treatment of latent tuberculosis infection. This official statement of the American Thoracic Society was adopted by the ATS Board of Directors, July 1999. This is a Joint Statement of the American Thoracic Society (ATS) and the Centers for Disease Control and Prevention (CDC). This statement was endorsed by the Council of the Infectious Diseases Society of America. (IDSA), September 1999, and the sections of this statement. Am J Respir Crit Care Med. 2000;161:S221–47. 10764341 10.1164/ajrccm.161.supplement_3.ats600

[11] Mazurek GH, Jereb J, Vernon A, LoBue P, Goldberg S, Castro K; IGRA Expert Committee; Centers for Disease Control and Prevention (CDC). Updated guidelines for using Interferon Gamma Release Assays to detect *Mycobacterium tuberculosis* infection - United States, 2010. MMWR Recomm Rep. 2010;59(RR-5):1–25. 20577159

[12] Powell DA, Perkins L, Wang SH, Hunt G, Ryan-Wenger N. Completion of therapy for latent tuberculosis in children of different nationalities. Pediatr Infect Dis J. 2008;27:272–4. 10.1097/INF.0b013e3181609a0a 18277917

[13] Cruz AT, Starke JR. Increasing adherence for latent tuberculosis infection therapy with health department-administered therapy. Pediatr Infect Dis J. 2012;31:193–5. 10.1097/INF.0b013e318236984f 21979799

[14] Gomes VF, Wejse C, Oliveira I, Andersen A, Vieira FJ, Carlos LJ, et al. Adherence to isoniazid preventive therapy in children exposed to tuberculosis: a prospective study from Guinea-Bissau. Int J Tuberc Lung Dis. 2011;15:1637–43. 10.5588/ijtld.10.0558 22118171

[15] Saunders MJ, Koh GC, Small AD, Dedicoat M. Predictors of contact tracing completion and outcomes in tuberculosis: a 21-year retrospective cohort study. Int J Tuberc Lung Dis. 2014;18:640–6. 10.5588/ijtld.13.0486 24903932

[16] Sloot R, Schim van der Loeff MF, Kouw PM, Borgdorff MW. Yield of tuberculosis contact investigations in Amsterdam: opportunities for improvement. Eur Respir J. 2014;44:714–24. 10.1183/09031936.00009114 25063246

[17] Turner RD, Chiu C, Churchyard GJ, Esmail H, Lewinsohn DM, Gandhi NR, et al. Tuberculosis infectiousness and host susceptibility. J Infect Dis. 2017;216(suppl_6):S636–43. 10.1093/infdis/jix361 29112746 PMC5853924

[18] Dheda K, Gumbo T, Maartens G, Dooley KE, McNerney R, Murray M, et al. The epidemiology, pathogenesis, transmission, diagnosis, and management of multidrug-resistant, extensively drug-resistant, and incurable tuberculosis. Lancet Respir Med. 2017;S2213-2600(17)30079-6. 28344011 10.1016/S2213-2600(17)30079-6

[19] Donald PR, Diacon AH, Lange C, Demers AM, von Groote-Biddlingmeier F, Nardell E. Droplets, dust and guinea pigs: an historical review of tuberculosis transmission research, 1878-1940. Int J Tuberc Lung Dis. 2018;22:972–82. 10.5588/ijtld.18.0173 30092861

[20] Van Wyk SS, Mandalakas AM, Enarson DA, Gie RP, Beyers N, Hesseling AC. Tuberculosis contact investigation in a high-burden setting: house or household? Int J Tuberc Lung Dis. 2012;16:157–62. 10.5588/ijtld.11.0393 22236914 PMC11969572

[21] Mandalakas AM, Kirchner HL, Lombard C, Walzl G, Grewal HM, Gie RP, et al. Well-quantified tuberculosis exposure is a reliable surrogate measure of tuberculosis infection. Int J Tuberc Lung Dis. 2012;16:1033–9. 10.5588/ijtld.12.0027 22692027 PMC11967560

[22] Garfein RS, Liu L, Cuevas-Mota J, Collins K, Muñoz F, Catanzaro DG, et al. Tuberculosis treatment monitoring by video directly observed therapy in 5 health districts, California, USA. Emerg Infect Dis. 2018;24:1806–15. 10.3201/eid2410.180459 30226154 PMC6154139

[23] Villarino ME, Scott NA, Weis SE, Weiner M, Conde MB, Jones B, et al.; International Maternal Pediatric and Adolescents AIDS Clinical Trials Group; Tuberculosis Trials Consortium. Treatment for preventing tuberculosis in children and adolescents: a randomized clinical trial of a 3-month, 12-dose regimen of a combination of rifapentine and isoniazid. JAMA Pediatr. 2015;169:247–55. 10.1001/jamapediatrics.2014.3158 25580725 PMC6624831

